# A Fractional Order Model Studying the Role of Negative and Positive Attitudes towards Vaccination

**DOI:** 10.3390/vaccines10122135

**Published:** 2022-12-13

**Authors:** Isa Abdullahi Baba, Fathalla A. Rihan, Usa Wannasingha Humphries, Badamasi Bashir Mikailu

**Affiliations:** 1 Department of Mathematics, Faculty of Science, King Mongkuts University of Science and Technology Thonburi (KMUTT), Bangkok 10140, Thailand; 2 Department of Mathematics, Bayero University Kano, Kano 700006, Nigeria; 3 Department of Mathematical Sciences, College of Science, UAE University, Al Ain 15551, United Arab Emirates; 4 Department of Mathematics, Faculty of Science, Helwan University, Cairo 11795, Egypt

**Keywords:** mathematical model, fractional-order, Caputo–Fabrizio, existence and uniqueness, vaccination, awareness

## Abstract

A fractional-order model consisting of a system of four equations in a Caputo–Fabrizio sense is constructed. This paper investigates the role of negative and positive attitudes towards vaccination in relation to infectious disease proliferation. Two equilibrium points, i.e., disease-free and endemic, are computed. Basic reproduction ratio is also deducted. The existence and uniqueness properties of the model are established. Stability analysis of the solutions of the model is carried out. Numerical simulations are carried out and the effects of negative and positive attitudes towards vaccination areclearly shown; the significance of the fractional-order from the biological point of view is also established. The positive effect of increasing awareness, which in turn increases positive attitudes towards vaccination, is also shown numerically.The results show that negative attitudes towards vaccination increase infectious disease proliferation and this can only be limited by mounting awareness campaigns in the population. It is also clear from our findings that the high vaccine hesitancy during the COVID-19 pandemicisan important problem, and further efforts should be madeto support people and give them correct information about vaccines.

## 1. Introduction

Scientific discoveries and their applications are what define modern societies. Recently, the emergence of anti-scientific attitudeshas led to a decrease in public trust in science [1]. Vaccines areamong the most significant discoveries in science, and have saved many lives. However, the increases anti-vaccine groups leads to vaccine rejection [2,3,4]. Hence, theseanti-vaccine groups increase the danger of infectious disease proliferation to themselves and to the entire society. Since the emergence ofthe COVID-19 pandemic and the serious problems it has caused, the study of the problemsleading to vaccine rejection is of paramount significance. Many people from different backgrounds are against vaccines, which consequently leads to reductions inpre-existing immunity [5].

Studying the causes of both negative and positive attitudes towards vaccination is therefore very significant as the purely scientific and applied perspectives are concerned. Several studies have investigated the causes of the increases in anti-vaccine groups and their focus has beengeared towards individual differencesmost of the time.For example, in [6] they claim that anti-vaccine attitudes are related tomoral purity concerns, and orthodox religiousness. In [7] they claim anti-vaccine attitudes have direct relationships withindividualistic/hierarchical worldviews and conspiratorial thinking.

Many models in theliterature have considered the vaccination decision-making process [8,9,10,11]. Most of these models are based on ordinary differential equations. In most of these models, the factors considered to affect the acceptance of vaccine are beliefs, fear, factual information, and rumors. Because of the hereditary properties and provision of a good description of the memory, fractional order derivatives and fractional integrals play important roles in mathematical modeling. This is why many researchers studyingreal-life phenomena use fractional order differential equations [12,13,14,15]. The Caputo–Fabrizio (CF) fractional-order derivative was developed in 2015. This fractional-order derivative is based on an exponential kernel and the details on the operator can be found in [16]. Many studies haveused the Caputo–Fabrizio derivative to model problems in various fields [17,18,19] and the Caputo–Fabrizio fractional derivative gives less noise than the Riemann–Liouville derivative [20]. Hence, in this research, the Caputo–Fabrizio fractional derivative was used.The Caputo-Fabrizio (CF) fractional operator used in this study is a particular case of the new generalized Hattaf fractional (GHF) operator presented in [21,22].

As for vaccine hesitancy, an investigation conducted in October 2020 [23] suggests that46%of French citizens are vaccine hesitant. Other countries exhibit percentages of opposition and hesitancy that exceed30%, i.e., 36%in Spain and the USA,35%in Italy,32%in South Africa, and31%in Japan and Germany. Globally, vaccinehesitancy and objection rates are as large as 27%.

Given these large percentages of hesitance and opposition to COVID-19 vaccines, [24] constructed a mathematical model that employs a behavioral epidemiology approach to study the implementation of a vaccination campaign for COVID-19. To this end, they adopt a strategy similar to the one used in [25]. In other words, they assume that the vaccination rate is a phenomenological function of the present and past information that the citizens have on the spread of the epidemic. Refs. [26,27,28,29] constructed a mathematical model in the context of SIR and SEIR infectious diseases, but thesemechanisticmodelsare based on evolutionary game theories which are reduced to the case of volatile opinion switching. Most of these models are either statistical or classical in nature, however in modeling hesitancy memory plays a vital role, hence the need for a fractional order model. Furthermore, the effect of awareness will be of paramount importance in reducing hesitancy. Our model is fractional in nature and also discusses the effect of awareness.

In our research, the main goal is to study the problem from a group processes viewpoint. In order to achieve this, we considered amathematical modeling approach. The target is to study the most significant parameters that lead to increases in anti-vaccine sentiments, and possibly study theeffects as they lead to increases in infectious disease proliferation using fractional order models. Present day social psychologists acknowledge that the most important procedure for group development is recognizing with the group and devotion to the group operation. Identity of a group can be seen from a cognitive-motivational perspective and the perspective of intergroup relations [8].

Here, we construct a model based on the Caputo–Fabrizio fractional derivative to study the role of negative and positive attitudes towards vaccination in relation to infectious disease proliferation. We divide the susceptible population into two:a pro-vaccination susceptible compartment and an anti-vaccination susceptible compartment. We also consider the possibility of changing compartments among the susceptible population, possibly due to change of mind.

## 2. Formulation of the Model

The model consists of a system of fractional order differential equations in the Caputo–Fabrizio sense with four compartments. The compartments are F(t),A(t),I(t), and R(t), which stand for the pro-vaccine susceptible compartment, the anti-vaccine susceptible compartment, the infected compartment, and the recovered compartment, respectively. The model is given below:

D0CFtαF(t)=−λαF(t)I(t)+p[μ1αI(t)+μ2αR(t)]−vαF(t)+γαA(t)−ηαF(t),D0CFtαA(t)=−λαA(t)I(t)+(1−p)[μ1αI(t)+μ2αR(t)]−γαA(t)+ηαF(t),D0CFtαI(t)=λα[F(t)+A(t)]I(t)−(βα+μ1α)I(t),D0CFtαR(t)=βαI(t)−μ2αR(t)+vαF(t),

with the following initial conditions:

$$F\left(0\right)=a_{1},A\left(0\right)=a_{2},I\left(0\right)=a_{3},\mathrm{and}\;R\left(0\right)=a_{4}.$$

Define N=F+A+I+R, to be the total population.

The meaning of the parameters involved in the model is given in Table 1 below.

## 3. Analysis of the Model

Here, existence and uniqueness analysis of the solution of the model iscarried out. Moreover, equilibria, basic reproduction number, and local stability analysis of the solution of the model are studied.

### 3.1. Existence and Uniqueness of a Solution of the Model

In this paper, a fixed-point result is applied to check the existence and uniqueness of the solution of the model. Let the system be re-written as

(1) D0CFtαF(t)=F1(t,F),

(2) D0CFtαA(t)=F2(t,A), 

(3) D0CFtαI(t)=F3(t,I),

(4) D0CFtαR(t)=F4(t,R). 

Applying the Caputo–Fabrizio operator, the system becomes:

(5) $$F\left(t\right)-F\left(0\right)=\frac{2\left(1-\alpha \right)}{\left(2-\alpha \right)M\left(\alpha \right)}F_{1}\left(t,F\right)+\frac{2\alpha }{\left(2-\alpha \right)M\left(\alpha \right)}\overset{t}{\underset{0}{\int }}F_{1}\left(\eta ,F\right)d\eta ,$$

(6) $$A\left(t\right)-A\left(0\right)=\frac{2\left(1-\alpha \right)}{\left(2-\alpha \right)M\left(\alpha \right)}F_{2}\left(t,A\right)+\frac{2\alpha }{\left(2-\alpha \right)M\left(\alpha \right)}\overset{t}{\underset{0}{\int }}F_{2}\left(\eta ,A\right)d\eta ,$$

(7) $$I\left(t\right)-I\left(0\right)=\frac{2\left(1-\alpha \right)}{\left(2-\alpha \right)M\left(\alpha \right)}F_{3}\left(t,I\right)+\frac{2\alpha }{\left(2-\alpha \right)M\left(\alpha \right)}\overset{t}{\underset{0}{\int }}F_{3}\left(\eta ,I\right)d\eta ,$$

(8) $$R\left(t\right)-R\left(0\right)=\frac{2\left(1-\alpha \right)}{\left(2-\alpha \right)M\left(\alpha \right)}F_{4}\left(t,R\right)+\frac{2\alpha }{\left(2-\alpha \right)M\left(\alpha \right)}\overset{t}{\underset{0}{\int }}F_{4}\left(\eta ,R\right)d\eta .$$

Now, we need to prove $F_{1},\ldots ,\;F_{4}$ satisfy Lipschitz continuity and contraction. See the theorem below:

**Theorem** **1.** $F_{1}$*is Lipschitz and if*

$$0\leq \lambda^{\alpha }k_{1}+v^{\alpha }+\eta^{\alpha }<1,$$

*it is a contraction.*

**Proof.**  $$\left\|F_{1}\left(t,F\right)-F_{1}\left(t,F^{1}\right)\|=\|-\lambda^{\alpha }I\left(t\right)\left(F\left(t\right)-F^{1}\left(t\right)\right)-\left(v^{\alpha }+\eta^{\alpha }\right)\left(F\left(t\right)-F^{1}\left(t\right)\right)\right\|\;\leq \lambda^{\alpha }\|I\left(t\right)\|\|F\left(t\right)-F^{1}\left(t\right)\|+\left(v^{\alpha }+\eta^{\alpha }\right)\|F\left(t\right)-F^{1}\left(t\right)\|\;\leq \left(\lambda^{\alpha }k_{1}+v^{\alpha }+\eta^{\alpha }\right)\|F\left(t\right)-F^{1}\left(t\right)\|\;\leq L_{1}\|F\left(t\right)-F^{1}\left(t\right)\|,$$

where $L_{1}=\lambda^{\alpha }k_{1}+v^{\alpha }+\eta^{\alpha }andk_{1}\geq \|I\left(t\right)\|.$

In the same way, we show the Lipschitz continuity and contraction for $F_{2},\ldots ,\;F_{4},$ where we obtain $L_{2},\ldots ,\;L_{4}$, respectively, as their Lipschitz constants.

In recursive form, let

(9) $$q_{1n}\left(t\right)=F^{n}\left(t\right)-F^{n-1}\left(t\right)=\frac{2\left(1-\alpha \right)}{\left(2-\alpha \right)M\left(\alpha \right)}\left(F_{1}\left(t,F^{n-1}\right)-F_{1}\left(t,F^{n-2}\right)\right)+\frac{2\alpha }{\left(2-\alpha \right)M\left(\alpha \right)}\overset{t}{\underset{0}{\int }}\left(F_{1}\left(\delta ,F^{n-1}\right)-F_{1}\left(\delta ,F^{n-2}\right)\right)d\delta ,$$

(10) $$q_{2n}\left(t\right)=A_{n}\left(t\right)-A_{n-1}\left(t\right)=\frac{2\left(1-\alpha \right)}{\left(2-\alpha \right)M\left(\alpha \right)}\left(F_{2}\left(t,A_{n-1}\right)-F_{2}\left(t,A_{n-2}\right)\right)+\frac{2\alpha }{\left(2-\alpha \right)M\left(\alpha \right)}\overset{t}{\underset{0}{\int }}\left(F_{2}\left(\delta ,A_{n-1}\right)-F_{2}\left(\delta ,A_{n-2}\right)\right)d\delta ,$$

(11) $$q_{3n}\left(t\right)=I_{n}\left(t\right)-I_{n-1}\left(t\right)=\frac{2\left(1-\alpha \right)}{\left(2-\alpha \right)M\left(\alpha \right)}\left(F_{3}\left(t,I_{n-1}\right)-F_{3}\left(t,I_{n-2}\right)\right)+\frac{2\alpha }{\left(2-\alpha \right)M\left(\alpha \right)}\overset{t}{\underset{0}{\int }}\left(F_{3}\left(\delta ,I_{n-1}\right)-F_{3}\left(\delta ,I_{n-2}\right)\right)d\delta ,$$

(12) $$q_{4n}\left(t\right)=R_{n}\left(t\right)-R_{n-1}\left(t\right)=\frac{2\left(1-\alpha \right)}{\left(2-\alpha \right)M\left(\alpha \right)}\left(F_{4}\left(t,R_{n-1}\right)-F_{4}\left(t,R_{n-2}\right)\right)+\frac{2\alpha }{\left(2-\alpha \right)M\left(\alpha \right)}\overset{t}{\underset{0}{\int }}\left(F_{4}\left(\delta ,R_{n-1}\right)-F_{4}\left(\delta ,R_{n-2}\right)\right)d\delta ,$$

with initial conditions:

(13) $$F_{0}\left(t\right)=F\left(0\right),A_{0}\left(t\right)=A\left(0\right),I_{0}\left(t\right)=I\left(0\right)andR_{0}\left(t\right)=R\left(0\right).$$

Taking norm of $q_{1n}$, we have:

(14) $$\|q_{1n}\left(t\right)\|=\|F^{n}\left(t\right)-F^{n-1}\left(t\right)\|\;=\|\frac{2\left(1-\alpha \right)}{\left(2-\alpha \right)M\left(\alpha \right)}\left(F_{1}\left(t,F^{n-1}\right)-F_{1}\left(t,F^{n-2}\right)\right)+\frac{2\alpha }{\left(2-\alpha \right)M\left(\alpha \right)}\overset{t}{\underset{0}{\int }}\left(F_{1}\left(\delta ,F^{n-1}\right)-F_{1}\left(\delta ,F^{n-2}\right)\right)d\delta \|.$$

Applying triangular inequality, we have:

$$\left\|q_{1n}\left(t\right)\|=\|F^{n}\left(t\right)-F^{n-1}\left(t\right)\|\;=\frac{2\left(1-\alpha \right)}{\left(2-\alpha \right)M\left(\alpha \right)}\|\left(F_{1}\left(t,F^{n-1}\right)-F_{1}\left(t,F^{n-2}\right)\right)\|+\frac{2\alpha }{\left(2-\alpha \right)M\left(\alpha \right)}\|\overset{t}{\underset{0}{\int }}\left(F_{1}\left(\delta ,F^{n-1}\right)-F_{1}\left(\delta ,F^{n-2}\right)\right)d\delta \right\|\;\leq \frac{2\left(1-\alpha \right)}{\left(2-\alpha \right)M\left(\alpha \right)}L_{1}\|F^{n}\left(t\right)-F^{n-1}\left(t\right)\|+\frac{2\alpha }{\left(2-\alpha \right)M\left(\alpha \right)}L_{1}\overset{t}{\underset{0}{\int }}\|F^{n}\left(t\right)-F^{n-1}\left(t\right)\|d\delta .$$

This implies:

(15) $$\|q_{1n}\left(t\right)\|\leq \frac{2\left(1-\alpha \right)}{\left(2-\alpha \right)M\left(\alpha \right)}L_{1}\|q_{1n-1}\left(t\right)\|+\frac{2\alpha }{\left(2-\alpha \right)M\left(\alpha \right)}L_{1}\overset{t}{\underset{0}{\int }}\|q_{1n-1}\left(t\right)\|d\delta .$$

Similarly,

(16) $$\|q_{2n}\left(t\right)\|\leq \frac{2\left(1-\alpha \right)}{\left(2-\alpha \right)M\left(\alpha \right)}L_{2}\|q_{2n-1}\left(t\right)\|+\frac{2\alpha }{\left(2-\alpha \right)M\left(\alpha \right)}L_{2}\overset{t}{\underset{0}{\int }}\|q_{2n-1}\left(t\right)\|d\delta ,$$

(17) $$\|q_{3n}\left(t\right)\|\leq \frac{2\left(1-\alpha \right)}{\left(2-\alpha \right)M\left(\alpha \right)}L_{3}\|q_{3n-1}\left(t\right)\|+\frac{2\alpha }{\left(2-\alpha \right)M\left(\alpha \right)}L_{3}\overset{t}{\underset{0}{\int }}\|q_{3n-1}\left(t\right)\|d\delta ,$$

(18) $$\|q_{4n}\left(t\right)\|\leq \frac{2\left(1-\alpha \right)}{\left(2-\alpha \right)M\left(\alpha \right)}L_{4}\|q_{4n-1}\left(t\right)\|+\frac{2\alpha }{\left(2-\alpha \right)M\left(\alpha \right)}L_{4}\overset{t}{\underset{0}{\int }}\|q_{4n-1}\left(t\right)\|d\delta .$$

Subsequently, we have:

(19) $$F_{n}\left(t\right)=\overset{n}{\underset{i=1}{\sum }}q_{1i}\left(t\right),\;A_{n}\left(t\right)=\overset{n}{\underset{i=1}{\sum }}q_{2i}\left(t\right),\;I_{n}\left(t\right)=\overset{n}{\underset{i=1}{\sum }}q_{3i}\left(t\right),\;R_{n}\left(t\right)=\overset{n}{\underset{i=1}{\sum }}q_{4i}\left(t\right).\;\;$$

□

To show the existence of the solution, we prove the following theorem:

**Theorem** **2.** *The solution exists if there exist* $t_{1}$ *such that the following inequality is true,*

(20) $$\frac{2\left(1-\alpha \right)}{\left(2-\alpha \right)M\left(\alpha \right)}L_{i}+\frac{2\alpha t_{1}}{\left(2-\alpha \right)M\left(\alpha \right)}L_{i}<1,\;i=1,\;\ldots ,\;5.\;$$

**Proof.**  Recursively, we have

(21) $$\|q_{1n}\left(t\right)\|\leq \|F^{n}\left(0\right)\|{\left[\frac{2\left(1-\alpha \right)}{\left(2-\alpha \right)M\left(\alpha \right)}L_{1}+\frac{2\alpha }{\left(2-\alpha \right)M\left(\alpha \right)}L_{1}\right]}^{n},\;$$

(22) $$\|q_{2n}\left(t\right)\|\leq \|A_{n}\left(0\right)\|{\left[\frac{2\left(1-\alpha \right)}{\left(2-\alpha \right)M\left(\alpha \right)}L_{2}+\frac{2\alpha }{\left(2-\alpha \right)M\left(\alpha \right)}L_{2}\right]}^{n},$$

(23) $$\|q_{3n}\left(t\right)\|\leq \|I_{n}\left(0\right)\|{\left[\frac{2\left(1-\alpha \right)}{\left(2-\alpha \right)M\left(\alpha \right)}L_{3}+\frac{2\alpha }{\left(2-\alpha \right)M\left(\alpha \right)}L_{3}\right]}^{n},\;$$

(24) $$\|q_{4n}\left(t\right)\|\leq \|R_{n}\left(0\right)\|{\left[\frac{2\left(1-\alpha \right)}{\left(2-\alpha \right)M\left(\alpha \right)}L_{4}+\frac{2\alpha }{\left(2-\alpha \right)M\left(\alpha \right)}L_{4}\right]}^{n}.\;$$

Hence, solutions exist and are continuous. To show that the functions above construct the solutions, consider:

(25) $$F\left(t\right)-F\left(0\right)=F^{n}\left(t\right)-H_{1_{n}}\left(t\right),\;$$

(26) $$A\left(t\right)-A\left(0\right)=A_{n}\left(t\right)-H_{2_{n}}\left(t\right),\;$$

(27) $$I\left(t\right)-I\left(0\right)=I_{n}\left(t\right)-H_{3_{n}}\left(t\right),$$

(28) $$R\left(t\right)-R\left(0\right)=R_{n}\left(t\right)-K_{4_{n}}\left(t\right).\;$$

Hence,

$$\left\|H_{1_{n}}\left(t\right)\|=\|\frac{2\left(1-\alpha \right)}{\left(2-\alpha \right)M\left(\alpha \right)}\left(F_{1}\left(t,F^{n-1}\right)-F_{1}\left(t,F^{n-2}\right)\right)+\frac{2\alpha }{\left(2-\alpha \right)M\left(\alpha \right)}\overset{t}{\underset{0}{\int }}\left(F_{1}\left(\delta ,F^{n-1}\right)-F_{1}\left(\delta ,F^{n-2}\right)\right)d\delta \right\|\;\leq \frac{2\left(1-\alpha \right)}{\left(2-\alpha \right)M\left(\alpha \right)}\|F_{1}\left(t,F^{n-1}\right)-F_{1}\left(t,F^{n-2}\right)\|+\frac{2\alpha }{\left(2-\alpha \right)M\left(\alpha \right)}\|\overset{t}{\underset{0}{\int }}\left(F_{1}\left(\delta ,F^{n-1}\right)-F_{1}\left(\delta ,F^{n-2}\right)\right)d\delta \|\;\leq \frac{2\left(1-\alpha \right)}{\left(2-\alpha \right)M\left(\alpha \right)}L_{1}\|F-F^{n-1}\|+\frac{2\alpha }{\left(2-\alpha \right)M\left(\alpha \right)}L_{1}\|F-F^{n-1}\|t.$$

Carrying out the procedure, we get

$$\|H_{1_{n}}\left(t\right)\|\leq {\left[\frac{2\left(1-\alpha \right)}{\left(2-\alpha \right)M\left(\alpha \right)}+\frac{2\alpha t}{\left(2-\alpha \right)M\left(\alpha \right)}\right]}^{n+1}L_{1}{}^{n+1}h.$$

At $t=t_{1},$ we get

$$\|H_{1_{n}}\left(t\right)\|\leq {\left[\frac{2\left(1-\alpha \right)}{\left(2-\alpha \right)M\left(\alpha \right)}+\frac{2\alpha t_{1}}{\left(2-\alpha \right)M\left(\alpha \right)}\right]}^{n+1}L_{1}{}^{n+1}h.$$

Taking limit as n→∞, we get

$$\|H_{1_{n}}\left(t\right)\|\to 0.$$

Similarly, we get

$$\|H_{2_{n}}\left(t\right)\|,\|H_{3_{n}}\left(t\right)\|,\;\|H_{4_{n}}\left(t\right)\|\to 0.$$

Finally, to show uniqueness, assume there exists some solutions say, $F^{1}\left(t\right),A^{1}\left(t\right),\;I^{1}\left(t\right)\;\mathrm{and}\;R^{1}\left(t\right),\;$ then

$$\|F\left(t\right)-F^{1}\left(t\right)\|\left(1-\frac{2\left(1-\alpha \right)}{\left(2-\alpha \right)M\left(\alpha \right)}L_{1}-\frac{2\alpha t}{\left(2-\alpha \right)M\left(\alpha \right)}L_{1}\right)\leq 0.$$

□

The following theorem completes the result.

**Theorem** **3.** *If*

$$\left(1-\frac{2\left(1-\alpha \right)}{\left(2-\alpha \right)M\left(\alpha \right)}L_{1}-\frac{2\alpha t}{\left(2-\alpha \right)M\left(\alpha \right)}L_{1}\right)>0,$$

*then the solution is unique.*

**Proof.**  Consider

$$\|F\left(t\right)-F^{1}\left(t\right)\|\left(1-\frac{2\left(1-\alpha \right)}{\left(2-\alpha \right)M\left(\alpha \right)}L_{1}-\frac{2\alpha t}{\left(2-\alpha \right)M\left(\alpha \right)}L_{1}\right)\leq 0.$$

Since,

$$\left(1-\frac{2\left(1-\alpha \right)}{\left(2-\alpha \right)M\left(\alpha \right)}L_{1}-\frac{2\alpha t}{\left(2-\alpha \right)M\left(\alpha \right)}L_{1}\right)>0,$$

then

$$\|F\left(t\right)-F^{1}\left(t\right)\|=0.$$

This implies,

$$F\left(t\right)=F^{1}\left(t\right).$$

□

This applies to the remaining functions.

Since R=N−(F+A+I), we can limit our analysis tothree compartments, {F,A,I}.

### 3.2. Equilibria and Basic Reproduction Number

The equilibrium solutions are obtained by equating the equations in the model to zero and solving the system simultaneously. We obtain two equilibrium solutions:

- Disease-free equilibrium ($E_{0}$)
  $$E_{0}=\left\{F_{0},\;A_{0},I_{0}\right\}=\left\{\frac{\lambda^{\alpha }\mu_{2}^{\alpha }N}{\left[\gamma^{\alpha }+\beta^{\alpha }+\left(1-p\right)v^{\alpha }\right]\mu_{2}^{\alpha }+\lambda^{\alpha }v^{\alpha }},\frac{\left[\eta^{\alpha }+\left(1-p\right)v^{\alpha }\right]\mu_{2}^{\alpha }N}{\left[\gamma^{\alpha }+\beta^{\alpha }+\left(1-p\right)v^{\alpha }\right]\mu_{2}^{\alpha }+\lambda^{\alpha }v^{\alpha }},0\right\}.$$
- Endemic equilibrium ($E_{1}$)
  $$E_{1}=\left\{F_{1},A_{1},I_{1}\right\},$$
  where
  $$F_{1}=\mu_{2}^{\alpha }\frac{\left(\frac{\lambda^{\alpha }N}{\beta^{\alpha }+\mu_{1}^{\alpha }}-1\right)}{v\frac{\lambda^{\alpha }}{\beta^{\alpha }+\mu_{1}^{\alpha }}}-\frac{\beta^{\alpha }+\mu_{2}^{\alpha }}{v^{\alpha }}I_{1},$$
  $$A_{1}=\frac{\beta^{\alpha }+\mu_{1}^{\alpha }}{\lambda^{\alpha }}-\left[\mu_{2}^{\alpha }\frac{\left(\frac{\lambda^{\alpha }N}{\beta^{\alpha }+\mu_{1}^{\alpha }}-1\right)}{v\frac{\lambda^{\alpha }}{\beta^{\alpha }+\mu_{1}^{\alpha }}}-\frac{\beta^{\alpha }+\mu_{2}^{\alpha }}{v^{\alpha }}I_{1}\right],$$
  and $I_{1}$ is obtained by solving the following quadratic equation,
  $$I_{1}=\frac{-a+\sqrt{a^{2}-bc}}{2b},$$
  where
  $$a=-\lambda^{\alpha }\mu_{2}^{\alpha }\frac{\left(\frac{\lambda^{\alpha }N}{\beta^{\alpha }+\mu_{1}^{\alpha }}-1\right)}{v\frac{\lambda^{\alpha }}{\beta^{\alpha }+\mu_{1}^{\alpha }}}+p\left(\mu_{1}^{\alpha }-\mu_{2}^{\alpha }\right)+\frac{\beta^{\alpha }+\mu_{2}^{\alpha }}{v^{\alpha }}\left(\gamma^{\alpha }+\eta^{\alpha }+v^{\alpha }\right),\;b=\lambda^{\alpha }\left(\frac{\beta^{\alpha }+\mu_{2}^{\alpha }}{v^{\alpha }}\right),\;and\;c=\frac{\gamma^{\alpha }\left(\beta^{\alpha }+\mu_{1}^{\alpha }\right)}{\lambda^{\alpha }}-\mu_{2}^{\alpha }\frac{\left(\frac{\lambda^{\alpha }N}{\beta^{\alpha }+\mu_{1}^{\alpha }}-1\right)}{v\frac{\lambda^{\alpha }}{\beta^{\alpha }+\mu_{1}^{\alpha }}}\left[\gamma^{\alpha }+\eta^{\alpha }+\left(1-p\right)v^{\alpha }\right].$$

Clearly, we can see that the endemic equilibrium exists only if,

$$\left(\frac{\lambda^{\alpha }N}{\beta^{\alpha }+\mu_{1}^{\alpha }}\right)\frac{\left[\gamma^{\alpha }+\eta^{\alpha }+\left(1-p\right)v^{\alpha }\right]\mu_{2}^{\alpha }}{\left[\gamma^{\alpha }+\eta^{\alpha }+\left(1-p\right)v^{\alpha }\right]\mu_{2}^{\alpha }+\gamma^{\alpha }v^{\alpha }}>1.$$

Define $\left(\frac{\lambda^{\alpha }N}{\beta^{\alpha }+\mu_{1}^{\alpha }}\right)\frac{\left[\gamma^{\alpha }+\eta^{\alpha }+\left(1-p\right)v^{\alpha }\right]\mu_{2}^{\alpha }}{\left[\gamma^{\alpha }+\eta^{\alpha }+\left(1-p\right)v^{\alpha }\right]\mu_{2}^{\alpha }+\gamma^{\alpha }v^{\alpha }}=R_{0},$ where $R_{0}$ is the basic reproduction number.

### 3.3. Local Stability Analysis of the Solution of the Model

Consider the following Jacobian matrix from (1),

(29) $$J=\left[\begin{array}{ccc} -\lambda^{\alpha }I-v^{\alpha }-\eta^{\alpha } & \Upsilon^{\alpha } & -\lambda^{\alpha }F+p\mu_{1}^{\alpha }\\ \eta^{\alpha } & -\lambda^{\alpha }I-\Upsilon^{\alpha } & -\lambda^{\alpha }A+\left(1-p\right)\mu_{1}^{\alpha }\\ \lambda^{\alpha }I & \lambda^{\alpha }I & \lambda^{\alpha }\left(F+A\right)-\left(\beta^{\alpha }+\mu_{1}^{\alpha }\right) \end{array}\right].$$

**Theorem** **4.** *The disease-free equilibrium is locally asymptotically stable if $R_{0}<1.$*

**Proof.**  Consider (29) at $E_{0}$, we get

(30) $$J\left(E_{0}\right)=\left[\begin{array}{ccc} -v^{\alpha }-\eta^{\alpha } & \Upsilon^{\alpha } & -\lambda^{\alpha }F_{0}+p\mu_{1}^{\alpha }\\ \eta^{\alpha } & -\Upsilon^{\alpha } & -\lambda^{\alpha }A_{0}+\left(1-p\right)\mu_{1}^{\alpha }\\ 0 & 0 & \lambda^{\alpha }\left(F_{0}+A_{0}\right)-\left(\beta^{\alpha }+\mu_{1}^{\alpha }\right) \end{array}\right].\;$$

The characteristics polynomial of (30) is

$$\left[-\eta^{\alpha }\Upsilon^{\alpha }+\left(-\left(v^{\alpha }+\eta^{\alpha }\right)-\lambda \right)\left(-\Upsilon^{\alpha }-\lambda \right)\right]\left(\lambda^{\alpha }\left(F_{0}+A_{0}\right)-\left(\beta^{\alpha }+\mu_{1}^{\alpha }\right)-\lambda \right)=0.$$

Therefore,

$$\lambda_{1}=\lambda^{\alpha }\left(F_{0}+A_{0}\right)-\left(\beta^{\alpha }+\mu_{1}^{\alpha }\right).$$

$\lambda_{2}\;\mathrm{and}\;\lambda_{3}$ can be found by solving $-\eta^{\alpha }\Upsilon^{\alpha }+\left(-\left(v^{\alpha }+\eta^{\alpha }\right)-\lambda \right)\left(-\Upsilon^{\alpha }-\lambda \right)=0.$

It is clear that $\lambda_{1}<0,\;if\lambda^{\alpha }\left(F_{0}+A_{0}\right)-\left(\beta^{\alpha }+\mu_{1}^{\alpha }\right)<0.$

This implies that $\frac{\lambda^{\alpha }\left(F_{0}+A_{0}\right)}{\left(\beta^{\alpha }+\mu_{1}^{\alpha }\right)}<1.$

Substituting the values of $F_{0}\;\mathrm{and}\;A_{0}$, we get

$$\left(\frac{\lambda^{\alpha }N}{\beta^{\alpha }+\mu_{1}^{\alpha }}\right)\frac{\left[\gamma^{\alpha }+\eta^{\alpha }+\left(1-p\right)v^{\alpha }\right]\mu_{2}^{\alpha }}{\left[\gamma^{\alpha }+\eta^{\alpha }+\left(1-p\right)v^{\alpha }\right]\mu_{2}^{\alpha }+\gamma^{\alpha }v^{\alpha }}=R_{0}<1.$$

Simplifying $-\eta^{\alpha }\Upsilon^{\alpha }+\left(-\left(v^{\alpha }+\eta^{\alpha }\right)-\lambda \right)\left(-\Upsilon^{\alpha }-\lambda \right)=0,$ we get

$$\lambda^{2}+\left(v^{\alpha }+\eta^{\alpha }+\Upsilon^{\alpha }\right)\lambda +v^{\alpha }\Upsilon^{\alpha }=0.$$

Using the Routh–Hurwitz criterion, $P\left(s\right)=s^{2}+a_{1}s+a_{2}$ has both roots with negative real parts iff both coefficeints, $a_{i}>0,\;i=0,1,2.$ Here,

$$a_{0}=1>0,\;a_{1}=\left(v^{\alpha }+\eta^{\alpha }+\Upsilon^{\alpha }\right)>0,\;ada_{2}=v^{\alpha }\Upsilon^{\alpha }>0.$$

Hence, $E_{0}$ is locally asymptotically stable if $R_{0}<1.$ □

**Theorem** **5.** *The endemic equilibrium is locally asymptotically stable if* $R_{0}>1,$ *and the following conditions are satisfied;*

$$i)\;\frac{p\mu_{1}^{\alpha }\lambda^{\alpha }I_{1}+\Upsilon^{\alpha }+\lambda^{\alpha }I_{1}+\beta^{\alpha }+\mu_{1}^{\alpha }}{\eta^{\alpha }+{\left(\lambda^{\alpha }\right)}^{2}I_{1}F_{1}+\lambda^{\alpha }\left(F_{1}+A_{1}\right)}>1,\;and\;ii)\frac{2{\left(\lambda^{\alpha }\right)}^{2}I_{1}A_{1}+\eta^{\alpha }\left[\lambda^{\alpha }\left(F_{1}+A_{1}\right)-\left(\beta^{\alpha }+\mu_{1}^{\alpha }\right)\right]+p\mu_{1}^{\alpha }\lambda^{\alpha }I_{1}\left(\eta^{\alpha }\lambda^{\alpha }+\Upsilon^{\alpha }+\lambda^{\alpha }I_{1}\right)}{{\left(\lambda^{\alpha }\right)}^{2}\left(\lambda^{\alpha }I_{1}+1\right)I_{1}F_{1}+2\left(1-p\right)\mu_{1}^{\alpha }\lambda^{\alpha }I_{1}+\left(\lambda^{\alpha }\left(F_{1}+A_{1}\right)-\left(\beta^{\alpha }+\mu_{1}^{\alpha }\right)\right)\left(\Upsilon^{\alpha }+\lambda^{\alpha }I_{1}\right)}>1.$$

**Proof.**  Consider (29) at $E_{1},$ we get

(31) $$J\left(E_{1}\right)=\left[\begin{array}{ccc} -\lambda^{\alpha }I_{1}-v^{\alpha }-\eta^{\alpha } & \Upsilon^{\alpha } & -\lambda^{\alpha }F_{1}+p\mu_{1}^{\alpha }\\ \eta^{\alpha } & -\lambda^{\alpha }I_{1}-\Upsilon^{\alpha } & -\lambda^{\alpha }A_{1}+\left(1-p\right)\mu_{1}^{\alpha }\\ \lambda^{\alpha }I_{1} & \lambda^{\alpha }I_{1} & \lambda^{\alpha }\left(F_{1}+A_{1}\right)-\left(\beta^{\alpha }+\mu_{1}^{\alpha }\right) \end{array}\right].\;$$

The characteristics polynomial of (31) is

$$\begin{array}{c} {}[\lambda^{2}+\lambda \left(-\eta^{\alpha }-{\left(\lambda^{\alpha }\right)}^{2}I_{1}F_{1}+p\mu_{1}^{\alpha }\lambda^{\alpha }I_{1}+\Upsilon^{\alpha }+\lambda^{\alpha }I_{1}-\lambda^{\alpha }\left(F_{1}+A_{1}\right)+\left(\beta^{\alpha }+\mu_{1}^{\alpha }\right)\right)\\ +(2{\left(\lambda^{\alpha }\right)}^{2}I_{1}A_{1}+\eta^{\alpha }\left[\lambda^{\alpha }\left(F_{1}+A_{1}\right)-\left(\beta^{\alpha }+\mu_{1}^{\alpha }\right)\right]+p\mu_{1}^{\alpha }\lambda^{\alpha }I_{1}\left(\eta^{\alpha }\lambda^{\alpha }+\Upsilon^{\alpha }+\lambda^{\alpha }I_{1}\right)\\ -\left({\left(\lambda^{\alpha }\right)}^{2}\left(\lambda^{\alpha }I_{1}+1\right)I_{1}F_{1}+2\left(1-p\right)\mu_{1}^{\alpha }\lambda^{\alpha }I_{1}+\left(\lambda^{\alpha }\left(F_{1}+A_{1}\right)-\left(\beta^{\alpha }+\mu_{1}^{\alpha }\right)\right)\left(\Upsilon^{\alpha }+\lambda^{\alpha }I_{1}\right)\right))][-\lambda^{\alpha }I_{1}\\ -v^{\alpha }-\eta^{\alpha }]=0. \end{array}$$

Therefore,

$$\lambda_{1}=-\lambda^{\alpha }I_{1}-v^{\alpha }-\eta^{\alpha }.$$

Clearly, $\lambda_{1}<0,$ if $I_{1}\geq 0,$ and $I_{1}\geq 0$ if $R_{0}>1.$

Applying the Routh–Hurwitz criterion to,

$$\begin{array}{c} \lambda^{2}+\lambda \left(-\eta^{\alpha }-{\left(\lambda^{\alpha }\right)}^{2}I_{1}F_{1}+p\mu_{1}^{\alpha }\lambda^{\alpha }I_{1}+\Upsilon^{\alpha }+\lambda^{\alpha }I_{1}-\lambda^{\alpha }\left(F_{1}+A_{1}\right)+\left(\beta^{\alpha }+\mu_{1}^{\alpha }\right)\right)\\ +(2{\left(\lambda^{\alpha }\right)}^{2}I_{1}A_{1}+\eta^{\alpha }\left[\lambda^{\alpha }\left(F_{1}+A_{1}\right)-\left(\beta^{\alpha }+\mu_{1}^{\alpha }\right)\right]+p\mu_{1}^{\alpha }\lambda^{\alpha }I_{1}\left(\eta^{\alpha }\lambda^{\alpha }+\Upsilon^{\alpha }+\lambda^{\alpha }I_{1}\right)\\ -\left({\left(\lambda^{\alpha }\right)}^{2}\left(\lambda^{\alpha }I_{1}+1\right)I_{1}F_{1}+2\left(1-p\right)\mu_{1}^{\alpha }\lambda^{\alpha }I_{1}+\left(\lambda^{\alpha }\left(F_{1}+A_{1}\right)-\left(\beta^{\alpha }+\mu_{1}^{\alpha }\right)\right)\left(\Upsilon^{\alpha }+\lambda^{\alpha }I_{1}\right)\right))=0, \end{array}$$

we see that the remaining Eigen values are negative if,

$$\frac{p\mu_{1}^{\alpha }\lambda^{\alpha }I_{1}+\Upsilon^{\alpha }+\lambda^{\alpha }I_{1}+\beta^{\alpha }+\mu_{1}^{\alpha }}{\eta^{\alpha }+{\left(\lambda^{\alpha }\right)}^{2}I_{1}F_{1}+\lambda^{\alpha }\left(F_{1}+A_{1}\right)}>1,$$

and

$$\frac{2{\left(\lambda^{\alpha }\right)}^{2}I_{1}A_{1}+\eta^{\alpha }\left[\lambda^{\alpha }\left(F_{1}+A_{1}\right)-\left(\beta^{\alpha }+\mu_{1}^{\alpha }\right)\right]+p\mu_{1}^{\alpha }\lambda^{\alpha }I_{1}\left(\eta^{\alpha }\lambda^{\alpha }+\Upsilon^{\alpha }+\lambda^{\alpha }I_{1}\right)}{{\left(\lambda^{\alpha }\right)}^{2}\left(\lambda^{\alpha }I_{1}+1\right)I_{1}F_{1}+2\left(1-p\right)\mu_{1}^{\alpha }\lambda^{\alpha }I_{1}+\left(\lambda^{\alpha }\left(F_{1}+A_{1}\right)-\left(\beta^{\alpha }+\mu_{1}^{\alpha }\right)\right)\left(\Upsilon^{\alpha }+\lambda^{\alpha }I_{1}\right)}>1.\;$$

□

## 4. Numerical Simulation

The numerical method used in this paper is similar to that of [21] and numerical simulations are carried out. Parameter values are given as, $\Lambda =0.6{\mathrm{day}}^{-1},\beta =0.5{\mathrm{day}}^{-1},\mu_{1}=0.001{\mathrm{day}}^{-1},\mu_{2}=0.0195{\mathrm{day}}^{-1},v=0.0005{\mathrm{day}}^{-1},\;\gamma =0.1{\mathrm{day}}^{-1},\;\eta =0.05{\mathrm{day}}^{-1},\;\alpha =0.2–1.0\left(\mathrm{dimentioneless}\right),p\;=0.5\left(\mathrm{dimentioneless}\right).$

**Figure 1:**
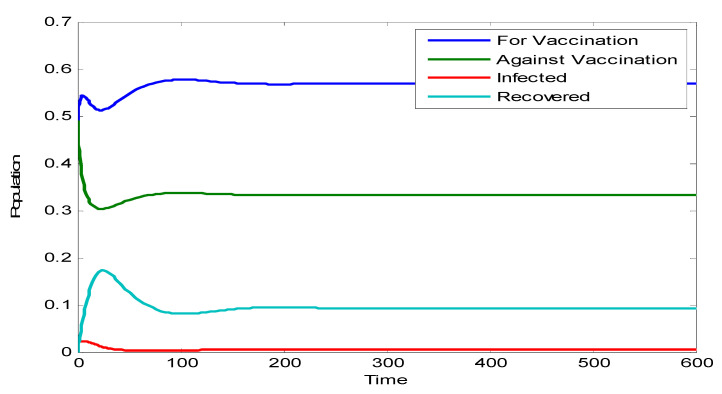
Dynamics of the model.

The dynamics of the model are depicted in Figure 1. It is clear that none of the populations go to zero. The infected population and the recovered population simultaneously reach their peak at around 50 h, which is approximately two days.

**Figure 2:**
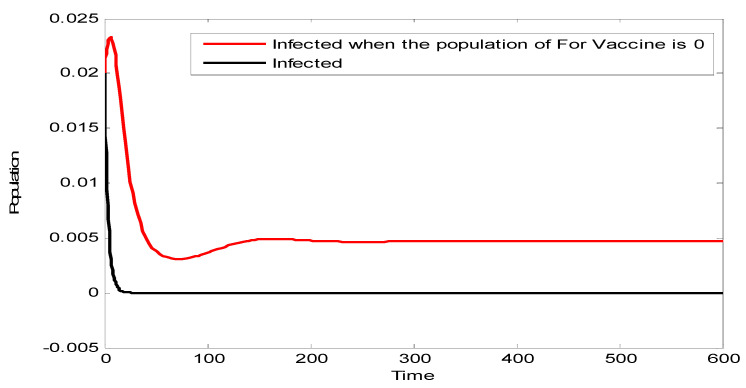
Effect of eliminating the pro-vaccine population.

Figure 2 compares the population of infected individuals with the pro-vaccine population. It can be seen that, in the absence of the pro-vaccine population, the infected population increases. This is because the remaining people in the population are against the vaccine and hence a large portion of the population will not be vaccinated. This leads to the proliferation of the disease.

**Figure 3:**
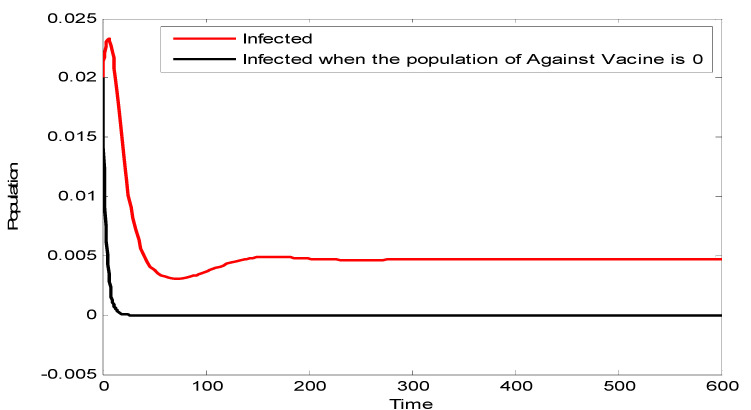
Effect of eliminating the anti-vaccine population.

Figure 3 compares the population of infected individuals with the anti-vaccine population. It can be seen that, in the absence of the anti-vaccine population, the infected population decreases. This is because the remaining people in the population are in support of the vaccine and hence a large portion of the population will be vaccinated. This leads to curtailing of the disease.

**Figure 4:**
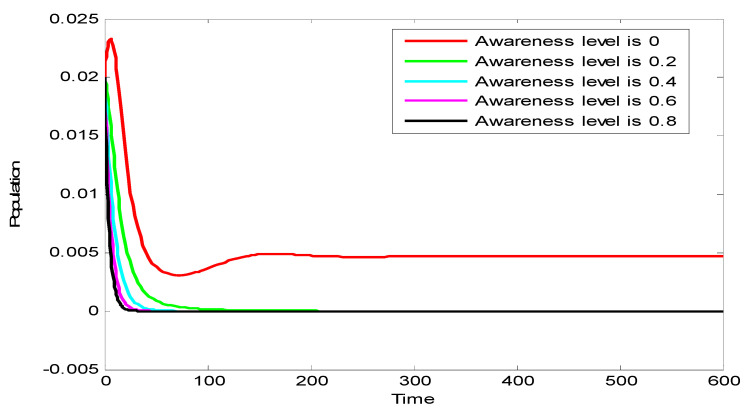
Effect of increasing awareness level.

Figure 4 shows that increase in the level of awareness lead to decreases in the population of infected individuals. This is because as the level of awareness increases, the number of pro-vaccine individuals increases. This leads to increases in the number of vaccinated individuals, which in turn leads to decreases in the population of infected individuals.

**Figure 5:**
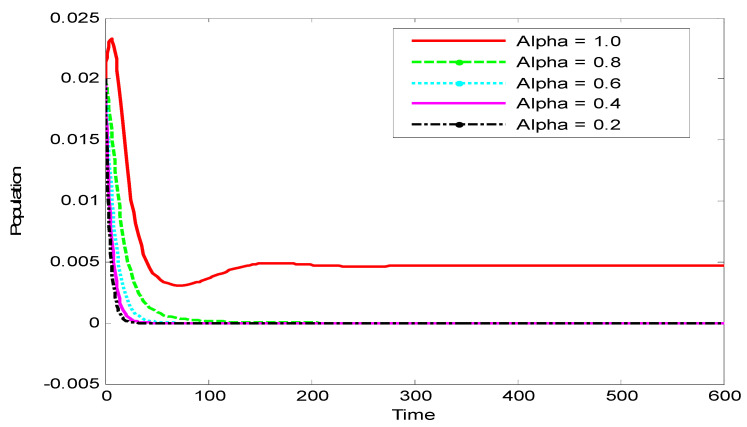
Dynamics of the infected population for various values of α.

Figure 5 shows the influence of the variation in the fractional-order α on the biological behavior of the infected population. It is clear from this figure that the population has an increasing effect when α is increased from 0.2 to 1.

## 5. Conclusions

In this paper, we studied a fractional-order model consisting of a system of four equations in the Caputo–Fabrizio sense. Our aim was to study the role of negative and positive attitudes towards vaccination in relation to infectious disease proliferation. The compartments of the model were the pro-vaccine susceptible compartment, the anti-vaccine susceptible compartment, the infected compartment, and the recovered compartment. We obtained two equilibrium solutions, i.e., disease free and endemic. We were also able to obtain the basic reproduction ratio. This paper studied the existence and uniqueness properties of the model in detail. Numerical simulations were carried out to support the analytic results. The effect of negative and positive attitudes towards vaccination was clearly shown. Furthermore, the significance of the fractional-order from the biological point of view was established. It was shown that increases in the level of awareness lead to decreases in the population of infected individuals. This is because as the level of awareness increases, the number of pro-vaccine individuals increases. This leads to increases in the number of vaccinated individuals, which in turn leads to decreases in the population of infected individuals.

The limitation of this study is that there is a need for real data collection to validate the model, and people’s opinions need to be heard and incorporated into the model for further analysis.

## Figures and Tables

**Table 1:** Meaning of parameters.

Variable/Parameter	Meaning
F	For-Vaccination susceptible compartment
A	Against-Vaccination susceptible compartment
I	Infected compartment
R	Recovered compartment
Λ	Infection rate
β	Recovery rate
$\mu_{1}$	Death rate of I
$\mu_{2}$	Death rate of R
v	Immunization rate of For-Vaccination compartment
γ	Migration rate from Against-Vaccination to For-Vaccination compartment through awareness
η	Migration rate from For-Vaccination to Against-Vaccination compartment through receiving false information about vaccines
α	Fractional order 0<α≤1
p	Probability term 0≤p≤1
